# Combination of *CLEC4M* rs868875 G-Carriership and *ABO* O Genotypes May Predict Faster Decay of FVIII Infused in Hemophilia A Patients

**DOI:** 10.3390/jcm11030733

**Published:** 2022-01-29

**Authors:** Barbara Lunghi, Massimo Morfini, Nicola Martinelli, Silvia Linari, Giancarlo Castaman, Francesco Bernardi

**Affiliations:** 1 Department of Life Sciences and Biotechnology, University of Ferrara, 44121 Ferrara, Italy; lngbbr@unife.it; 2 Italian Association Hemophilia Centers (AICE), 80131 Naples, Italy; drmassimomorfini@gmail.com; 3 Department of Medicine, University of Verona, 37134 Verona, Italy; nicola.martinelli@univr.it; 4 Center for Bleeding Disorders, Department of Oncology, Careggi University Hospital, 50134 Florence, Italy; linaris@aou-careggi.toscana.it (S.L.); castaman@aou-careggi.toscana.it (G.C.)

**Keywords:** *CLEC4M*, *CLEC4M* SNPs, factor VIII, haemophilia A, pharmacokinetics, *ABO*, half-life, clearance

## Abstract

The C-type lectin CLEC4M binds and internalizes factor VIII (FVIII). Common *CLEC4M* variants have been associated with FVIII pharmacokinetic (PK) profiles in hemophilia A (HA) patients. The two-compartment PK analysis of plasma-derived (pd-) and full length recombinant FVIII concentrates was conducted in twenty-six patients (FVIII:C ≤ 2 IU/dL). *F8*, *ABO* blood-groups, and the *CLEC4M* rs868875A/G polymorphism were genotyped. *CLEC4M* genotype groups differed for the elimination rate constant K 1-0 (*p* < 0.001), half-life (K 1-0 HL), and the Beta rate constant. Patients treated with pd-FVIII also differed in the Alpha phase. In linear regression models, the contribution of the *CLEC4M* genotypes to FVIII PK parameters remained significant after correction for *ABO*, age, and VWF antigen levels at PK. Combined *CLEC4M* rs868875A/G and *ABO* genotypes displayed significant interaction (K 1-0, *p* = 0.014). Compared to other combined genotypes, the G-carriers/O genotypes showed half-reduced K 1-0 HL (*p* = 0.008), and faster FVIII clearance (mean 7.1 ± 2.2 mL/h/kg SE) than in the G-carriers/non-O (mean 2.4 ± 0.3 mL/h/kg SE), (*p* = 0.038). Comparison in HA patients recruited in several countries suggests that *CLEC4M* genotypes coherently influence infused FVIII half-life and clearance. Our analysis supports substantially faster FVIII decay associated with the rs868875 G-carrier/*ABO* O genotypes, which has potential implications for genetically tailored substitutive HA treatment.

## 1. Introduction

Genetic components, together with environmental factors [1,2,3,4,5,6], provide explanation only for a small portion of the large inter-patient variability of factor VIII (FVIII) pharmacokinetics (PK) in hemophilia A (HA) patients. Age, von Willebrand factor (VWF) levels, and the ABO blood group altogether explain 30% of such variability in severe HA [1,2,3,4,5].

Among the candidate scavenger receptors [7] for circulating FVIII/VWF, the low-density lipoprotein receptor (*LDLR*), the stabilin 2 (*STAB2*), and the asialoglycoprotein receptor minor subunit gene (*ASGR2*) have been recently associated with FVIII pharmacokinetics (PK) through gene variation in small size HA cohorts [8,9,10], independently from *ABO* blood-group for the *LDLR* and *ASGR2* [8,10] genotypes.

Among receptors, the C-type lectin domain family 4 member M (CLEC4M), also termed L-SIGN or DC-SIGNR, is expressed on the sinusoidal endothelial cells of the liver, the main source of circulating FVIII, and thus, is of noticeable interest both for biological and clinical reasons. Its extra-cellular binding domain consists of an extended neck region, which contains tandem repeats, followed by a C-terminal C-type carbohydrate-recognition domain, which complexes carbohydrates of a high-mannose type, well represented in FVIII [11,12] and VWF molecules [13,14].

In cellular and animal models, CLEC4M binds and internalizes FVIII in a VWF-dependent and -independent manner [15]. CLEC4M can also act as a cell-adhesion and pathogen-recognition receptor, which could provide a link between coagulation and infection [16].

Common variants at the *CLEC4M* locus were associated with the FVIII PK profile in a population of severe pediatric HA patients, and in populations of adult moderate/severe HA patients [9,17,18]. These observations have been obtained by the infusion of different plasma-derived (pd-) or recombinant (r-) standard half-life FVIII concentrates, and by different PK analysis approaches, the PopPK [17], and the model-independent method individual PK [9,18].

Prompted by these observations, and aimed at understanding specific components of interest for substitutive treatment in HA patients, we have analyzed association of *CLEC4M* genotypes with two-compartment model full length (FL) FVIII PK parameters, and compared the degree of association with those observed in recently published studies. We focused on the interaction between *CLEC4M* gene variation and *ABO* genotypes, which are well recognized genetic components of FVIII PK outcomes [1,8,19,20].

## 2. Patients and Methods

### 2.1. Clinical Study

This study was conducted in accordance with the principles of Helsinki Declaration, and with Good Clinical Practice. This study reports an investigator-initiated IRB-approved retrospective chart review of FVIII PK performed in the Center for Bleeding Disorders, Careggi University Hospital, Florence, Italy, for optimization of replacement treatment. Patients expressed their oral and written informed consent for PK execution and genotyping, originally performed for F8 mutation detection.

### 2.2. Study Design and Patients

Patients with severe or moderately severe HA (FVIII:C ≤ 2 IU/dL), treated with plasma-derived (pd)-FVIII or r-FVIII SHL concentrates, were selected. Patients affected by full-blown AIDS (CD4 < 200/mcL) were not included in the study. Patients who had the FVIII inhibitor test by Nijmegen assay > 0.4 IU/dL or INR > 1.3 were excluded.

Twenty-six patients (median age 39 years, SD 14 years, range 14–67 years) were investigated for FVIII PK and *CLEC4M* genotype. The PK study was conducted as previously described [10]. After a 3–4 day wash out, HA patients were infused with a single dose of FVIII products (22.7–51.8 IU/kg). Twenty-two patients were treated with pd-FVIII, and eleven with r-FVIII concentrates. For seven patients, PKs were obtained with both FVIII concentrates. Twelve patients (46%) underwent repeated PKs (2–5).

### 2.3. Plasma Assays

Platelet poor plasma was stored at −40 °C in 0.5 mL aliquots. FVIII coagulant activity assays (One-Stage Method) were done at the same time on three duplicate dilutions of baseline and post-infusion samples [21]. VWF antigen (VWF:Ag) determination was performed as previously described [22], and was available for *n* = 24 patients.

### 2.4. PK Methods

The blood samples were collected before infusion and after 0.25, 0.5, 1, 3, 6, 9, 24, 28, 48, and 72 h, for better evaluation of the Beta phase. Each PK decay was analyzed according to the Two-Compartment Model (TCM) by WinNonlin 7.0 (Phoenix 64, Pharsight, Mountain View, CA, USA). From 54 PKs, resulted fitting the TCM better, we considered specific final and secondary PK parameters listed in the legend to Table 1. In the twelve patients who underwent repeated PKs, mean PK parameter values were calculated and used as single case values for association studies with genetic polymorphisms.

### 2.5. Polymorphisms and Genotyping

*F8* mutations were found by direct sequencing [23], F8 intron 22 inversion (IVS 22), and *ABO* blood-group, as previously described [2,24]. The rs868875 A/G polymorphism of *CLEC4M* gene was investigated by TaqI restriction analysis of a PCR fragment (215 bp) obtained by using the mutagenized forward primer (5′-GTGTGATGTGACTTTACTTGAGTTATC-3′) and the reverse primer (5′-AGGAGTCCTGGCTCCATCTCT-3′) that introduced a TaqI restriction site in the G allele (189 and 26 bp).

### 2.6. CLEC4M rs868875 A/G Genotypes and FVIII PK Parameters: A Literature Search

We compared the results obtained in the present study with three published studies: (i) Swystun et al. [9] reported the TCIWorks PK analysis of 43 pediatric HA patients infused with r-FVIII products; (ii) Garcia-Martinez et al. [17] reported the myPK-Fit PopPK analysis of 43 pediatric/adult HA patients infused with r-FVIII (ADVATE) products; (iii) Ogiwara et al. [18] reported the PKRD (PharmacoKinetics Repeated Doses) or the TCIWorks PK analysis of 43 adult HA patients infused with r-FVIII (80%) and pd-FVIII (20%) products.

PK parameter values reported for the most frequent rs868875 AA and AG genotypes [9,18] were compared in Italian patients. The constant for the elimination rate from the central compartment K 1-0 (1/h), K 1-0 half-life (K 1-0 HL, h), and clearance (mL/h) were compared with the following assumptions and/or limitations: (i) K, half-life, and clearance were according to TCIWorks [9,18] and myPK-Fit [17], whereas in the present study, K 1-0, K 1-0 HL, and clearance were according to the 2 CP model PK; (ii) in Garcia-Martinez et al. [17], the G-allele-related increments are reported for a single allele and for the homozygous GG condition; (iii) clearance data were compared after adjustment for a mean weight of 70 kg.

### 2.7. Statistical Analysis

All statistical analyses were performed using IBM^®^ SPSS^®^ Statistics version 23.0 software (IBM Corp., Armonk, NY, USA). Continuous variables with normal distribution (MRT and AUC) were expressed as means with standard error (SE). Skewed variables were logarithmically transformed, and means with SE were reported. Genotype-related differences in PK parameters were analyzed by *t*-test, Mann–Whitney test, or analysis of variance (ANOVA) for linear trend, as appropriate. The contribution to PK parameters of *CLEC4M* rs868875 (G-carriers vs. AA), *ABO* (O vs. non-O) genotypes, age, and VWF:Ag levels were evaluated by linear regression analysis, and the interaction between genotypes was estimated as *p* (int) by generalized linear model.

## 3. Results

The relationship between the *CLEC4M* rs868875 genotypes and FVIII PK parameters was investigated in the cohort of Italian HA patients (*n* = 26) infused with pd- and FL r-FVIII products (54 PKs).

In patients grouped by genotypes (AA, *n* = 12; AG, *n* = 12, and GG, *n* = 2), values of PK parameters displayed significant differences for the final elimination rate constant K 1-0 (*p* < 0.001, ANOVA for linear trend), for the transfer from the central to peripheral plasma compartment rate constant K 1-2 (*p* = 0.049), the secondary Beta (*p* = 0.030), and K 1-0 HL (*p* = 0.011). A trend was observed for the Beta HL (*p* = 0.054) (Table 1).

Patient carriers of the G allele (*n* = 14) showed K 1-0 values (0.11 ± 0.03 1/h SE) higher than AA homozygotes (0.06 ± 0.00 1/h SE, *p* = 0.045).

When the analysis was restricted to the patients who underwent at least one PK with a pd-FVIII (*n* = 22, Table S1), PK parameters in genotyped patients (AA, *n* = 9; AG, *n* = 11; GG, *n* = 2) displayed several and significant differences, also for the Alpha distribution phase (Alpha, *p* = 0.033; Alpha HL, *p* = 0.007).

Patients grouped by G-allele carriership (*n* = 13) showed K 1-0 HL values (mean 8.07 ± 0.92 h SE) 28% (approximately 3 h) shorter (*p* = 0.062) than AA-homozygotes (mean 11.2 ± 1.07 h SE).

### Combination of CLEC4M and ABO Genotypes

ABO blood groups are a well-known modulator of FVIII PK [1,8,19,20]. Accordingly, in the Italian cohort, the *ABO* genotype groups (O, non-O) showed significant differences for several parameters (Table S2).

We compared the influence on FVIII PK of the *CLEC4M* and *ABO* genotypes in linear regression models of PK variables associated with *CLEC4M* genotypes. The contribution of the *CLEC4M* rs868875 polymorphism remained significant for K 1-0, Beta, K 1-0 HL, and, as a trend, for Beta HL PK parameters (Table 2). For the Beta, Beta HL, and K 1-2, the contribution of the *ABO* genotypes prevailed (Table 2). We observed significant interaction between genotypes, particularly for K 1-0, K 1-0 HL, and Beta HL (Table 2).

The significant contribution of *CLEC4M* rs868875 A/G and *ABO* genotypes was maintained after inclusion in the model of age and VWF:Ag levels at PK (Table S3). The influence of age was observed as a trend for the Beta parameters.

VWF:Ag levels were not associated with *CLEC4M* G-carriership (AA, 135 ± 9.6% SE; AG + GG, 122 ± 7.3% SE).

**Figure 1:**
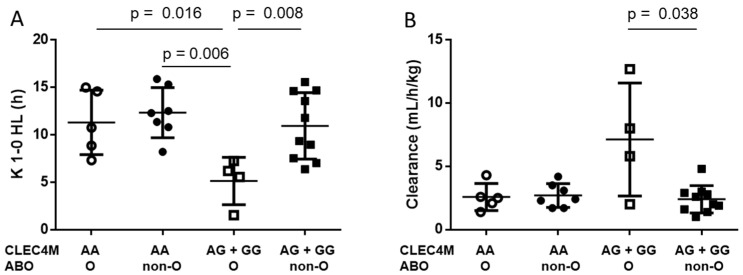
(**A**) K 1-0 HL and (**B**) clearance PK parameters in combined *CLEC4M* rs868875 A/G and *ABO* genotypes in the 26 HA patients treated with FL FVIII products. Mean values ± standard deviation are reported. *p*, Mann–Whitney test.

Prompted by these observations, we report the distribution of K 1-0 HL (Figure 1A) in the 26 patients grouped by combination of *CLEC4M* and *ABO* genotypes. The G-carriers/O blood group genotypes showed significantly shorter K 1-0 HL values than all the other genotypes groups. In contrast, in the AA homozygotes, the K 1-0 HL did not differ in relation to the *ABO* genotypes (Figure 1A).

To favor comparison with previous studies, we also analyzed the clearance distribution in the *CLEC4M*/*ABO* genotypes groups. Although with overlapping of values, mean clearance of infused FVIII was higher (*p* = 0.038) in the G-carriers/O blood group (mean 7.13 ± 2.23 mL/h/kg SE) than in the G-carriers/non-O (mean 2.40 ± 0.34 mL/h/kg SE) (Figure 1B).

## 4. Discussion

Previous studies have detected association of the *CLEC4M* rs868875 A/G polymorphism with clearance [9,17,18], and with half-life [17] FVIII PK parameters (Table 3), detected by different PK analysis approaches. Aimed at improving our knowledge of main components of the association between *CLEC4M* genotypes and FVIII PK parameters, which could provide useful information for HA patients’ treatment, we contribute a two compartment (2CP) model PK analysis of patients with severe/moderate HA.

We compared the association of the rs868875 *CLEC4M* genotypes with FVIII PK parameters in the different studies (Table 3), with reasonable assumptions permitting value paralleling. A substantial concordance of PK parameter distribution was observed in relation to the *CLEC4M* genotypes in HA patients, particularly for the elimination constant rate (K/K 1-0) and the half-life/K 1-0 HL. FVIII clearance was found to be 25–40% increased in G-carriers, a noticeable observation for the critical parameter to tailor prophylaxis or continuous infusion. This observation was coherent with the lower K/K 1-0 elimination rate constant, and with the shorter HL/K 1-0 HL values, albeit with one discrepancy (Ogiwara et al. [18], Table 3). A comparison of parameter values in the different studies suggests that the presence of at least one G-allele may shorten the FVIII half-life. We believe that the observation of similar results in several studies with small size HA cohorts strengthens these findings.

Differences in FVIII half-life could be amplified in adults, whereas those in clearance could be more prominent in children and young individuals [17].

In the Italian patients, linear regression analysis suggested both *ABO* and *CLEC4M* genotypes as significant predictors of FVIII-PK, which displayed significant interaction to influence the Beta and elimination rates, independently from age at PK. The effects of the G-allele carriership, a genetic approach that strengthens our findings, were particularly pronounced in patients belonging to the O blood group. The combined genotype *CLEC4M* rs868875 G-carrier/*ABO* O, detectable in 15% of patients, produced significantly shorter FVIII PK half-life and increased clearance, major results of our study. Since the mean FVIII half-life was approximately half-reduced as compared with all other combined genotypes, and was reflected in a largely increased clearance, this finding deserves to be explored for personalized replacement therapy/prophylaxis. Since our observations were obtained in a limited number of HA patients, and the infrequent *CLEC4M* rs868875 GG homozygous condition does not favor the exploration of the potentially higher influence of this genotype, confirmation in larger cohorts is needed. Provided that our findings were confirmed, the practicalities needed to implement the routine clinical practice in FVIII PK analysis in HA would not be particularly demanding in terms of genetic analysis, as in other diseases. Patients with the unfavorable genotype combination would benefit from infusion with a higher FVIII dose or extended half-life FVIII products.

It is worth noting that in cellular and animal models, CLEC4M binds and internalizes FVIII both in a VWF-dependent and -independent manner [15]. This would imply that the modulation on FVIII PK parameters by receptors encoded by different *CLEC4M* genotypes may be exerted both through binding to FVIII and to VWF–FVIII complex. As a consequence, studies aimed at disentangling FVIII- and VWF-specific PK components could be disfavored by the several genetic/acquired components modulating VWF expression, as supported by the absence of significant differences in VWF:Ag levels in patients grouped by *CLEC4M* genotypes in the present study, and in Swystun et al. and Garcia-Martinez et al. [9,17]. In linear regression analysis, the effects of the *CLEC4M* and *ABO* genetic components were independent from the VWF:Ag levels, which, however, are strongly associated with the *ABO* genotypes.

Concerning the FVIII concentrates used for the PK analysis, we observed a remarkable influence of *CLEC4M* genotypes on PK analysis with pd-FVIII products. Although it is tentative to speculate that “natural” glycosylation of FVIII favors interaction with the receptor, comparison with other studies, which used only recombinant products [9,17], suggests that *CLEC4M* genotypes influence the PK of FVIII concentrates characterized by differences in glycan structure after recombinant expression. This observation will favor further comparison with extended half-life FVIII concentrates, which will require appropriately designed studies.

## 5. Conclusions

Overall, observations in patients recruited in several countries support that specific *CLEC4M* genotypes influence half-life and clearance of different FVIII concentrates infused in HA patients. Our analysis suggests that the combined *CLEC4M* G-carrier and *ABO* O genotypes contribute to a faster decay of infused FVIII in HA, which deserves to be explored for personalized replacement therapy/prophylaxis.

## Figures and Tables

**Table 1:** Analysis of association between pd- and FL-recombinant FVIII PK parameters, and the *CLEC4M* rs868875 polymorphism.

PK Parameters	*CLEC4M* rs868875 Genotypes			
	AA (*n* = 12)	AG (*n* = 12)	GG (*n* = 2)	*p*
Final				
K 1-0 (1/h)	0.06 (±0.00)	0.08 (±0.01)	0.28 (±0.16)	**<0.001**
K 1-2 (1/h)	0.38 (±0.12)	0.36 (±0.13)	3.24 (±1.63)	**0.049**
K 2-1 (1/h)	0.85 (±0.21)	0.52 (±0.08)	1.76 (±0.20)	0.374
V1 (dL/kg)	0.43 (±0.04)	0.43 (±0.07)	0.21 (±0.08)	0.088
Secondary				
Alpha (1/h)	1.29 (±0.34)	0.92 (±0.20)	5.19 (±1.57)	0.127
Alpha HL (h)	2.39 (±0.76)	1.99 (±0.47)	0.17 (±0.07)	0.139
Beta (1/h)	0.04 (±0.00)	0.05 (±0.01)	0.08 (±0.02)	**0.030**
Beta HL (h)	19.0 (±2.22)	17.4 (±2.21)	9.12 (±2.37)	0.054
Cl (mL/h/kg)	2.58 (±0.31)	3.83 (±0.99)	4.30 (±1.70)	0.350
CLD2 (mL/h/kg)	15.1 (±0.05)	14.4 (±0.05)	48.0 (±0.16)	0.201
Cmax (IU/dL)	79.5 (±6.9)	98.7 (±13.4)	103 (±11.0)	0.209
K 1-0 HL (h)	11.9 (±0.83)	10.1 (±1.07)	4.30 (±2.74)	**0.011**
* MRT (h)	24.4 (±2.1)	22.8 (±2.8)	12.8 (±3.6)	0.148
* AUC (IU.h/dL)	1373 (±145)	1434 (±226)	525 (±268)	0.327
AUCM (IU.h2/dL)	35,886 (±6005)	38,565 (±9362)	7694 (±5316)	0.060

The mean values with standard error of continuous variables are reported. *, normally distributed variables. K 1-0, elimination rate constant from the central compartment; K 1-2, transfer rate constant from central (1) to peripheral (2) compartment; K 2-1, transfer rate constant from peripheral (2) to central (1) compartment; V1, volume of central compartment; Alpha, alpha rate constant associated with the initial distribution phase; Alpha HL, alpha distribution half-life; Beta, beta rate constant associated with the elimination phase; Beta HL, beta elimination half-life; Cl, clearance; CLD2, inter-compartmental clearance; Cmax, at zero time extrapolated FVIII:C concentration; K 1-0 HL, K 1-0 half-life; MRT, mean residence time; AUC, area under the curve; AUMC, the moment of AUC. *p*, ANOVA analysis, in bold, *p* < 0.05.

**Table 2:** Linear regression model for predictors of FVIII PK parameter variability.

PK Parameters	ß-Coefficient	*p*	Predictors/Genotypes	*p* (int)
K 1-0 (1/h)	0.457	**0.013**	G-carriers vs. AA	**0.014**
	−0.440	**0.016**	O vs. non-O	
K 1-2 (1/h)	0.243	0.211	G-carriers vs. AA	0.889
	−0.409	**0.040**	O vs. non-O	
Beta (h)	0.378	**0.031**	G-carriers vs. AA	0.071
	−0.546	**0.003**	O vs. non-O	
Beta HL (h)	−0.329	0.060	G-carriers vs. AA	**0.049**
	0.564	**0.002**	O vs. non-O	
K 1-0 HL (h)	−0.410	**0.028**	G-carriers vs. AA	**0.047**
	0.433	**0.021**	O vs. non-O	

G-carriers vs. AA, *CLEC4M* genotypes; O vs. non-O, *ABO* genotypes. K 1-0, elimination rate constant from the central compartment; K 1-0 HL, K 1-0 half-life; Beta, beta rate constant associated with the elimination phase; Beta HL, beta elimination half-life. *p*, regression analysis; *p* (int), interaction between genotypes, generalized linear model; in bold, *p* < 0.05.

**Table 3:** *CLEC4M* rs868875 genotypes and FVIII PK parameters.

	FVIII Products	*CLEC4M* Genotypes	K/K 1-0 (1/h)		Half-Life/ K 1-0 HL (h)		Clearance (mL/h)	
Swystun et al. Blood 2019	r-FVIII (100%)	AA	0.06	↑	11	↓	120	↑
		AG	0.07		9		180	↑
			*p* = ns		*p* = ns		*p* = 8.0 × 10^−3^	
Garcia-Martinez et al. TH 2020	r-FVIII (100%)	AA			−1.1	↓	+21 ^§^	↑
		AG			−2.2	↓	+42 ^§^	↑
		GG			*p* = 2.90 × 10^−5^		*p* = 1.01 × 10^−3^	
Ogiwara et al. JTH 2021		AA			10	↑	* 280	↑
	r-FVIII (80%)	AG			12		* 400	↑
	pd-FVIII (20%)	GG			11		300	
					*p* = ns		* *p* = 1.5 × 10^−2^	
Present study °	pd-FVIII ° (100%)	AA	* 0.07	↑	* 11.15	↓	* 154	↑
		AG	* 0.09	↑	* 8.75	↓	* 202	
		GG	0.28		4.3		268	
			*p* = 1.0 × 10^−3^		*p* = 2.0 × 10^−3^		*p* = ns	
			* *p* = 0.079		* *p* = 0.080		* *p* = ns	

Comparison of association between the *CLEC4M* rs868875 genotypes and FVIII PK parameters estimated from published studies. G-allele-related increments estimated from Garcia-Martinez et al. are reported. r-FVIII, recombinant FVIII; pd-FVIII, plasma derived FVIII concentrates. ° Values obtained with only pd-FVIII. ^§^ after adjustment for a mean weight of 70 kg. K, half-life, and clearance according to TCIWorks [9,18] and myPK-Fit [17]; K 1-0, K 1-0 HL, and clearance of the present study according to WinNonlin 2CP model. K 1-0, the elimination rate constant from the central compartment. For the present study: *p*, ANOVA; * *p*, *t*-test analysis. ns, not significant value. The arrows indicate decreased (↓) or increased (↑) values in relation to the AA genotypes, and are meant to quickly compare parameter values in the different PK studies. Double arrows, statistically significant association; single arrow, not significant (ns).

## Data Availability

Data supporting reported results can be obtained by request to Dr M. Morfini (drmassimomorfini@gmail.com).

## Supplementary Materials

The following are available online at: https://www.mdpi.com/article/10.3390/jcm11030733/s1, Table S1: Analysis of association between plasma derived FVIII PK parameters and the *CLEC4M* rs868875 polymorphism. Table S2: Analysis of association between FVIII PK parameters and the *ABO* blood group genotypes in the 26 severe/moderate HA patients. Table S3: Linear regression model for predictors of FVIII PK parameter variability.
