# Supplementary material for: Combination of CLEC4M rs868875 G-Carriership and ABO O Genotypes May Predict Faster Decay of FVIII Infused in Hemophilia A Patients
Source: J Clin Med. 2022 Jan 29;11(3):733. doi: 10.3390/jcm11030733 (PMC8837058; doi:10.3390/jcm11030733)
Supplement: Supplementary file 1 [file jcm-11-00733-s001.zip › jcm-1504717-supplementary.pdf]

Table S1. Analysis of association between plasma derived FVIII PK parameters and the *CLEC4M* rs868875 polymorphism.

**PK parameters**	***CLEC4M* rs868875 genotypes**			
	AA (n = 9)	AG (n = 11)	GG (n = 2)	p
**Final**				
K 1-0 (1/h)	0.07 (± 0.01)	0.09 (± 0.01)	0.28 (± 0.16)	**0.001**
K 1-2 (1/h)	0.35 (± 0.11)	0.42 (± 0.15)	3.24 (± 1.63)	**0.026**
K 2-1 (1/h)	0.70 (± 0.17)	0.67 (± 0.10)	1.76 (± 0.20)	0.103
V1 (dL/kg)	0.38 (± 0.04)	0.43 (± 0.08)	0.21 (± 0.08)	0.260
**Secondary**				
Alpha (1/h)	1.08 (± 0.27)	1.12 (± 0.22)	5.19 (± 1.57)	**0.033**
Alpha HL (h)	2.31 (± 0.83)	1.01 (± 0.20)	0.17 (± 0.07)	**0.007**
Beta (1/h)	0.04 (± 0.00)	0.06 (± 0.01)	0.08 (± 0.02)	**0.019**
Beta HL (h)	18.8 (± 2.80)	14.2 (± 1.90)	9.12 (± 2.37)	**0.018**
Cl (mL/h/kg)	2.54 (± 1.06)	3.97 (± 1.06)	4.20 (± 1.60)	0.280
CLD2 (mL/h/kg)	12.2 (± 4.44)	16.4 (± 5.76)	48.0 (± 0.16)	0.082
Cmax (IU/dL)	74.8 (± 8.3)	101 (± 15.8)	103 (± 11.0)	0.223
K 1-0 HL (h)	11.2 (± 1.07)	8.75 (± 0.87)	4.30 (± 2.74)	**0.002**
*MRT (h)	23.7 (± 2.6)	19.7 (± 2.7)	12.8 (± 3.6)	0.106
*AUC (IU.h/dL)	1198 (± 147)	1273 (± 233)	525 (± 268)	0.431
AUCM (IU.h^2/dL)	30971 (± 7044)	29501 (± 9245)	7694 (± 5316)	0.066

The mean values with standard error of continuous variables are reported. *, normally distributed variables. K 1-0, elimination rate constant from the central compartment; K 1-2, transfer rate constant from central (1) to peripheral (2) compartment; K 2-1, transfer rate constant from peripheral (2) to central (1) compartment; V1, Volume of central compartment. Alpha, alpha rate constant associated with the distribution phase; Alpha HL, alfa distribution half-life; Beta, beta rate constant associated with the elimination phase; Beta HL, beta elimination half-life; Cl, clearance; CLD2, inter-compartmental clearance; Cmax, at zero time extrapolated FVIII:C concentration; K 1-0 HL, K 1-0 half-life; MRT, mean residence time; AUC, area under the curve; AUMC, the moment of AUC. p, ANOVA analysis, in bold, $p < 0.05$.
K 1-0 values in AA vs AG genotypes, $p = 0.079$; K 1-0 HL values in AA vs AG genotypes, $p = 0.080$.

Table S2. Analysis of association between FVIII PK parameters and the *ABO* blood group genotypes in the 26 severe/moderate HA patients.

**PK parameters**	***ABO* genotypes**		
	O (*n* = 9)	non-O (*n* = 17)	*p*
**Final**			
K 1-0 (1/h)	0.12 (± 0.04)	0.07 (± 0.01)	0.057
K 1-2 (1/h)	0.96 (± 0.50)	0.40 (± 0.14)	0.058
K 2-1 (1/h)	1.04 (± 0.09)	0.63 (± 0.17)	**0.014**
V1 (dL/kg)	0.46 (± 0.09)	0.39 (± 0.03)	0.823
**Secondary**			
Alpha (1/h)	2.13 (± 0.60)	1.04 (± 0.30)	**0.014**
Alpha HL (h)	0.78 (± 0.18)	2.70 (± 0.58)	**0.035**
Beta (1/h)	0.06 (± 0.01)	0.04 (± 0.00)	**0.010**
Beta HL (h)	12.7 (± 1.50)	20.0 (± 1.91)	**0.007**
Cl (mL/h/kg)	4.60 (± 1.24)	2.52 (± 0.24)	0.066
CLD2 (mL/h/kg)	29.0 (± 7.96)	11.1 (± 3.76)	**0.022**
Cmax (IU/dL)	75.9 (± 10.3)	97.8 (± 9.07)	0.076
K 1-0 HL (h)	8.58 (± 1.44)	11.5 (± 0.77)	0.058
*MRT (h)	17.7 (± 2.2)	25.4 (± 2.1)	**0.027**
*AUC (IU.h/dL)	928 (± 203)	1552 (± 147)	**0.020**
AUCM (IU.h^2/dL)	19669 (± 6246)	43046 (± 6646)	**0.008**

The mean values with standard error of continuous variables are reported. *, normally distributed variables. K 1-0, elimination rate constant from the central compartment; K 1-2, transfer rate constant from central (1) to peripheral (2) compartment; K 2-1, transfer rate constant from peripheral (2) to central (1) compartment; V1, Volume of central compartment. Alpha, alpha rate constant associated with the distribution phase; Alpha HL, alfa distribution half-life; Beta, beta rate constant associated with the elimination phase; Beta HL, beta elimination half-life; Cl, clearance; CLD2, inter-compartmental clearance; Cmax, at zero time extrapolated FVIII:C concentration; K 1-0 HL, K 1-0 half-life; MRT, mean residence time; AUC, area under the curve; AUMC, the moment of AUC. *p*, ANOVA analysis, in bold, $p < 0.05$.

Table S3. Linear regression model for predictors of FVIII PK parameter variability.

PK parameters	ß-coefficient	*p*	Predictors/Genotypes
K 1-0 (1/h)	0.512	**0.010**	G-carriers vs. AA
	-0.601	**0.004**	O vs. non-O
	-0.266	0.152	age
	0.253	0.180	VWF:Ag
K 1-0 HL (h)	-0.428	**0.038**	G-carriers vs. AA
	0.555	**0.011**	O vs. non-O
	0.205	0.299	age
	-0.144	0.473	VWF:Ag
Beta (h)	0.369	**0.041**	G-carriers vs. AA
	-0.650	**0.001**	O vs. non-O
	-0.328	0.067	age
	-0.040	0.819	VWF:Ag
Beta HL (h)	-0.317	0.080	G-carriers vs. AA
	0.670	**0.001**	O vs. non-O
	0.307	0.090	age
	0.027	0.881	VWF:Ag

G-carriers vs. AA, *CLEC4M* genotypes; O vs. non-O, *ABO* genotypes. VWF antigen levels (VWF:Ag) were available for $n = 24$ patients. K 1-0, elimination rate constant from the central compartment; K 1-0 HL, K 1-0 half-life; Beta, beta rate constant associated with the elimination phase; Beta HL, beta elimination half-life. *p*, regression analysis; in bold, $p < 0.05$.
